# Breeding effects on durum wheat traits detected using GWAS and haplotype block analysis

**DOI:** 10.3389/fpls.2023.1206517

**Published:** 2023-09-19

**Authors:** F. Taranto, S. Esposito, F. Fania, R. Sica, S. Marzario, G. Logozzo, T. Gioia, P. De Vita

**Affiliations:** ^1^ Italian National Council of Research (CNR), Institute of Biosciences and Bioresources (IBBR), Bari, Italy; ^2^ Council for Agricultural Research and Economics (CREA), Research Centre for Cereal and Industrial Crops (CREA-CI), Foggia, Italy; ^3^ Department of Agriculture, Food, Natural Resources, and Engineering (DAFNE) - University of Foggia, Foggia, Italy; ^4^ School of Agricultural, Forestry, Food and Environmental Sciences, University of Basilicata, Potenza, Italy

**Keywords:** multi-locus GWAS, quantitative trait nucleotide (QTN), haplotype blocks, plant variety protection, UPOV protocol

## Abstract

**Introduction:**

The recent boosting of genomic data in durum wheat (*Triticum turgidum* subsp. *durum*) offers the opportunity to better understand the effects of breeding on the genetic structures that regulate the expression of traits of agronomic interest. Furthermore, the identification of DNA markers useful for marker-assisted selection could also improve the reliability of technical protocols used for variety protection and registration.

**Methods:**

Within this motivation context, 123 durum wheat accessions, classified into three groups: landraces (LR), ancient (OC) and modern cultivars (MC), were evaluated in two locations, for 34 agronomic traits, including UPOV descriptors, to assess the impact of changes that occurred during modern breeding.

**Results:**

The association mapping analysis, performed with 4,241 SNP markers and six multi-locus-GWAS models, revealed 28 reliable Quantitative Trait Nucleotides (QTNs) related to plant morphology and kernel-related traits. Some important genes controlling flowering time and plant height were in linkage disequilibrium (LD) decay with QTNs identified in this study. A strong association for yellow berry was found on chromosome 6A (*Q.Yb-6A*) in a region containing the *nadh-ubiquinone oxidoreductase* subunit, a gene involved in starch metabolism. The *Q.Kcp-2A* harbored the PPO locus, with the associated marker (*Ku_c13700_1196*) in LD decay with *Ppo-A1* and *Ppo-A2*. Interestingly, the *Q.FGSGls-2B.1*, identified by *RAC875_c34512_685* for flag leaf glaucosity, mapped less than 1 Mb from the *Epistatic inhibitors of glaucousness* (*Iw1*), thus representing a good candidate for supporting the morphological DUS traits also with molecular markers. LD haplotype block approach revealed a higher diversity, richness and length of haploblocks in MC than OC and LR (580 in LR, 585 in OC and 612 in MC), suggesting a possible effect exerted by breeding programs on genomic regions associated with the agronomic traits.

**Discussion:**

Our findings pave new ways to support the phenotypic characterization necessary for variety registration by using a panel of cost-effectiveness SNP markers associated also to the UPOV descriptors. Moreover, the panel of associated SNPs might represent a reservoir of favourable alleles to use in durum wheat breeding and genetics.

## Introduction

Durum wheat (*Triticum turgidum* subsp. *durum*) is a major staple crop in the Mediterranean basin, and Italy and Spain are among the main producers (Eurostat, 2023). The crop is mainly used to produce pasta, which is consumed not only in the Mediterranean regions but worldwide (Broccanello et al., 2023). In addition to its economic importance, durum wheat is also important for its nutritional value. It is high in protein and dietary fiber, and it contains essential vitamins and minerals that play a very important role in the prevention of diabetes and cardiovascular diseases (Garutti et al., 2022). To ensure the sustainability of the crop and increase its yield and quality, plant breeders constantly selected high-yielding varieties with improved grain quality traits. In Italy, more than 300 durum wheat varieties are currently registered to the National Register of Varieties by several representative institutions and companies (Sian, 2023), emphasizing the intense breeding work conducted in Italy over the last decades. In the past, numerous studies were conducted to evaluate the impact of durum wheat breeding on yield and grain quality and define a new plant ideotype capable of maximizing yields in different environmental conditions. The genetic gain was systematically evaluated by comparing the performances of historical varieties released over different points of breeding time (Pecetti and Annicchiarico, 1998; Motzo et al., 2004; De Vita et al., 2007), and the results showed a genetic gain for durum wheat of about 0.6% per year, similar to the value reported in many cases of bread wheat (Calderini and Slafer, 1999). The superiority of the modern wheat cultivars in terms of grain yield has been attributed largely to changes in the harvest index, with small or negligible increases in total biomass production and the number of grains per unit of land (Austin et al., 1980; Austin, 1989; Siddique et al., 1989; Slafer and Andrade, 1993; Brancourt-Hulmel et al., 2003). These findings demonstrate how modern breeding has been successful in exploiting crop diversity for genetic improvement in both bread and durum wheat. However, the recent estimates of genetic gain for both species are stagnant, probably because modern intensive breeding practices have exploited the residual fraction of the available crop diversity and/or as a result of climate change. The narrow genetic base of elite germplasm compromises long-term genetic gain and increases the genetic vulnerability to unpredictable environmental conditions.

Efficient genetic diversity management is therefore required in breeding programs and low-cost genotyping platforms that generate thousands to millions of data points provide effective means for crop genetic research studies (Ganal et al., 2012). For wheat, a large amount of single nucleotide polymorphisms (SNPs), generated using different genotyping platforms, was available thanks to the recent release of the high-quality reference genome of bread and durum wheat. This opened new possibilities for untangling the genetic architecture of complex traits by genome-wide association study (Rabbi et al., 2021; Eltaher et al., 2022; Esposito et al., 2022) and to perform other genomic studies, for instance, the analysis of selective sweeps within or across species (Liu et al., 2019; Semagn et al., 2021; Soriano et al., 2021). In addition, recent studies on wheat and other crops have shown that Genome-Wide Association Studies (GWAS) coupled with haplotypic block (HB) analysis improved analysis based on a single marker not only in terms of statistical significance (better p-values) but also in estimating the allelic effects (Hao et al., 2012; Lu et al., 2012; N’Diaye et al., 2017; Ledesma-Ramírez et al., 2019; Li et al., 2019; Sehgal and Dreisigacker, 2019; Shokat et al., 2020).

The genetic architecture of complex traits can provide insights into the underlying genetic mechanisms that control the expression of these traits and develop new cultivars with improved traits. Moreover, to guarantee the plant variety protections at the end of the selection process, the use of molecular markers to discriminate morphological traits could be used in distinctness, uniformity and stability (DUS) testing of new varieties as a complement to, or replacement of, morphological observations listed in International Union for the Protection of New Varieties of Plants (UPOV) guidelines (Jamali et al., 2019; Yu and Chung, 2021).

In our previous study (Taranto et al., 2020) a large panel of Italian durum wheat accessions that includes landraces (LR), old (OC) and modern cultivars (MC) was subjected to genotyping using the Illumina iSelect 15K wheat SNP array. The study was carried out with the aims of i) assessing the genetic diversity and population structure in a large collection of durum wheat accessions (over 250) released since the early 1900s using genome-wide high-density SNP array and ii) understanding the history of Italian durum wheat breeding by identifying molecular signatures of divergence and selection. Relatively small differences in genetic diversity were observed among accessions whereas an increase in linkage disequilibrium (LD) and in changes in the allelic frequencies in DNA regions that control important agronomic traits, were found. In the LD analysis, to exclude possible bias due to the different sizes of sub-population and to minimize the sampling effect, we standardized the number of accessions of LR, OC and MC to 41, for a total of 123 accessions. Using this balanced core set of genotypes, in this paper, we present the results obtained by performing GWAS and LD HB analysis on several durum wheat traits, including grain quality and yield-related traits, morphological characters described in the UPOV test guidelines (UPOV, 2012), and grain morphometric parameters. The goals were to: i) identify phenotype-genotype associations for the 34 traits analyzed by GWAS; ii) evaluate the breeding effect by analyzing the temporal trend of LD haplotype blocks moving from the landraces to modern cultivars; and iii) identify specific candidate genes for each of the 3 breeding groups (i.e., LR, OC, MC).

## Materials and methods

### Plant material and phenotyping

A core collection of 123 durum wheat genotypes was derived from a larger panel of accessions previously developed by Taranto et al. (2020), including 41 landraces, 41 old cultivars and 41 modern cultivars. Two experiments were carried out during the growing season 2018/2019 at the CREA, Research Centre for Cereal and Industrial Crops, Foggia, Italy (41’ 46° N, 16’54° E, altitude 70 m) and Metaponto (A.A.S.D. Pantanello of ALSIA, MT, Basilicata, 40° 23’ 27.7’’ N, 16° 47’ 15.1” E), respectively.

The list of phenotypic traits and their acronyms used afterward were reported in **Table S1**. For details of the agronomic protocols of the two experiments and the phenotypic traits analysis, see Marzario et al. (2023) submitted. In detail, in both locations the experiments were arranged in a randomized complete block with three replicates, in a field trial in Foggia and in a greenhouse experiment in Metaponto, respectively. Ten seeds for each accession in each replicate were sown in a single row plot (1 m long, 0.3 m apart). At maturity, ten main spikes with well-developed grains were randomly collected from each accession and replicates. The accessions were manually harvested and shelled to avoid seed contamination. The agronomic management of the crop was the most widespread in the area, while weeds were controlled manually. Phenotypic values were also combined to determine the best linear unbiased predictions (BLUPs) values to eliminate the environmental deviation and estimate the real individual breeding value. In particular, BLUPs were calculated using the ‘lme4’ package of the R4.0.1 software (www.r-project.org), with location as random effects in the model [Y = lmer (X~(1|LINE) + (1|LOC) + (1|LINE : LOC)] (Merk et al., 2012; Bates et al., 2015) to generate a precise estimation of genotypic values (Mi et al., 2011; Viana et al., 2011). Normal distribution of BLUP data was verified using the Kolmogorov-Smirnov (D), the Shapiro-Wilk (w) and Anderson-Darling (A) tests implemented in the stats R package (R Core Team, 2017). For all methods, the null hypothesis of normal distribution was accepted for traits showing p. values > 0.05.

### Genome-wide association analysis

The 123 accessions included in the durum wheat panel were genotyped by Taranto et al. (2020) using wheat Illumina 15K Infinium SNP array developed at the TraitGenetics (available online: http://www.traitgenetics.com). The Svevo v.1.0 reference genome was used to assign the physical position to each SNP marker (Maccaferri et al., 2019). A set of 8,491 SNPs were filtered with Plink (Purcell et al., 2007) using a call rate value lower than 95% and with a minimum allele frequency (MAF) lower than 5%. After filtering, a total number of 4,241 SNPs was used for the downstream analysis.

Association analyses were performed using the data derived from the single environments and validated with BLUPs. Six Multi-Locus (ML) GWAS models (mrMLM, FASTmrMLM, FASTmrEMMA, pLARmEB and ISIS EMBLASSO) implemented in the R package mrMLM v4.0 (Zhang et al., 2020) were used with default parameters. The genomic regions with LOD score of ≥ 3.00 were considered as Quantitative Trait Nucleotides (QTNs) significantly associated with the traits under study. Further, SNP markers that were repeatedly detected in both environments and confirmed by BLUPs values were designated as reliable associated QTNs.

### LD haplotype block analysis

To evaluate changes in the LD haplotype block during the Italian durum wheat breeding, the collection was divided into three groups (LR, OC and MC), according to the partition by Taranto et al. (2020). The LD haplotype blocks were detected using SVS software (version 8.8.1, Golden Helix Inc.), with the MAF threshold set to 0.01. The confidence interval algorithm was developed by Gabriel et al. (2002) and detailed in Sehgal et al. (2020). The minimum lower and upper confidence interval values were set to 0.75 and 0.90, respectively.

### Candidate genes

Candidate genes flanking the significant marker-trait associations’ (MTAs) regions were searched based on the LD haplotype blocks and LD decay distance calculated by Taranto et al. (2020). High-confidence genes along with their functional annotation and Gene Ontology (GO) terms were retrieved from Svevo durum genome v 1.0 (Maccaferri et al., 2019; http://www.gramene.org/) using Ensembl Plants (Cunningham et al., 2022; https://plants.ensembl.org/index.html) and biomart (Kinsella et al., 2011).

Significant QTNs were annotated using the Svevo durum wheat high-confidence gene models (Maccaferri et al., 2019). To determine which classes of genes were over-represented, the Gene Set Enrichment Analysis (GSEA, AgriGO v2. Analytical toolkit (Tian et al., 2017) was performed using the hypergeometric test and corrected via Hochberg FDR option. A False Discovery Rate (FDR) < 0.05 cut-off has been set for detecting significantly enriched groups. MapMan4 (Schwacke et al., 2019) was also employed to integrate and visualize the functions of candidate genes in metabolic pathways. The Svevo mapping file was created with MapMan Mercator (Lohse et al., 2014) and loaded into the software. Only pathways marked as X4 were used.

## Results

### Phenotypic distribution

The distributions of derived BLUPs values for all traits are reported in **Table S2** and **Figure S1**. Considering the Kolmogorov-Smirnov test, the null hypothesis of normal distribution could be accepted for all the traits (*p.value* > 0.05), with the exception of Kernel thickness (KT), Blackstain Blackpoints (BP) and Plant height (PH), which showed *p.value* < 0.05 (**Table S3**). Normality was also investigated with Shapiro-Wilk (w) and Anderson-Darling (A) tests, which confirmed normal distributions for Spikelets number/spike (SpktSPK), Number of kernels/spike (KerSPK), thousand kernels weight (TKW), Kernel width (KerWid), Grain Protein Content (GPC), Sedimentation SDS test (SDS), Total Carotenoid Content (TCC) and Awn length (AwnLen) (**Table S3**).

### Multi-locus GWAS

**Figure 1:**
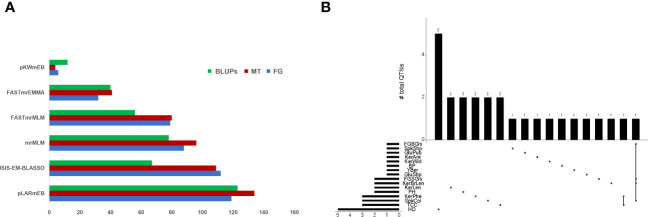
**(A)** Number of QTNs identified by each of the six ML-GWAS models in Foggia (FG), Metaponto (MT), and by using BLUP values. **(B)** UpSet plot showing the intersection of “reliable QTNs” identified by ML-GWAS. The number of QTNs is indicated for each intersection.

A total of 436, 464 and 376 QTNs associated with thirty-four traits (LOD score ≥ 3) were identified at Foggia (FG), Metaponto (MT) and by using BLUPs, respectively (**Figure 1A**). The highest number of QTNs was found using pLARmEB (119 in FG and 134 QTNs in MT), followed by ISIS-EM-BLASSO (112 in FG and 109 QTNs in MT), mrMLM (88 in FG and 96 QTNs in MT), FASTmrMLM (79 in FG and 80 QTNs in MT), FASTmrEMMA (32 in FG and 41 QTNs in MT) and pKWmEB (6 in FG and 4 QTNs in MT) (**Figure 1**). A total of 28 QTNs were declared as “reliable QTNs” since they were identified in both environments and confirmed by BLUPs (**Table 1**; **Figure 1B**). Among these latter, one QTN (*Q.Yber-6A*) was detected by all methods and it explained a phenotypic variance of up to 32%. Other eight QTNs (for PH, Yber, TCC, KerLen, FGSGls and GluShp) were identified by five models in all environments, with the only exception of pKWmEB which failed in detecting the associations. These QTNs showed LOD values up to 9 for TCC and KerLen and explained a phenotypic variance ranging between 13% for PH and 29% for the FGSGls.

**Table 1:** List of the 28 reliable QTNs identified for the agronomic traits detected by ML-GWAS.

Trait	QTN	SNP_ID	Chr	Position	Enviroment	LOD score	‘-log10(P)’	r^2^ (%)	GWA Method
Plant height	*Q.Ph-2B*	Kukri_c36879_83	2B	96408120	foggia	3,14-11,02	3,85-11,98	3,67-13,16	2,3,5,4
					metaponto	3,78-6,29	7,13-7,13	1,61-5,61	2,5,4
					combined	3,63-7,34	4,36-8,22	3,00-5,87	1,2,3,4,5
	*Q.Ph-4A*	Tdurum_contig31218_344	4A	733401525	foggia	3.88	4.62	2.63	5
					metaponto	4,55-6,44	5,33-7,28	2,64-3,07	2.5
					combined	3,24-6,12	3,95-6,96	1,91-2,45	1,3,5
Kernel area	*Q.KerAre-3B*	RAC875_rep_c83245_239	3B	486046320	foggia	4.43	5.20	6.94	5
					metaponto	7.15	8.02	7.78	4
					combined	4,68-9,64	5,57-10,57	4,65-8,69	4.5
Yellow berry	*Q.Yber-6A*	Excalibur_c9713_247	6A	47184856	foggia	3,18-6,85	3,88-7,71	4,68-16,48	1,2,3,4,5
					metaponto	4.33	5.10	7.81	2
					combined	3,70-7,44	4,77-8,32	5,11-32,80	1,2,3,4,5,6
Total Carotenoid Content	*Q.Tcc-2A*	BobWhite_c31163_694	2A	743587463	foggia	3,19-3,23	3,90-3,94	3,25-5,04	1.2
					metaponto	5,91-7,05	6,74-7,92	9,86-13,98	1.2
					combined	4,17-7,30	4,93-8,18	5,90-7,05	1,2,4,5
	*Q.Tcc-6B.1*	BobWhite_c1633_643	6B	645616232	foggia	3,44-6,61	4,16-7,46	3,28-9,22	1,2,3,5
					metaponto	4,67-5,28	5,46-6,08	5,34-9,08	1.2
					combined	3,25-4,72	3,97-5,51	3,38-15,07	1.4
	*Q.Tcc-6B.2*	Tdurum_contig11700_1247	6B	132017890	foggia	6,15-9,02	6,91-9,94	8,14-13,47	1,2,3,4,5
					metaponto	4,98-5,44	4,06-6,26	4,53-11,53	1,2,3,4
					combined	3,90-9,91	4,65-10,85	5,71-13,07	1,2,3,4,5
Kernel width	*Q.KerWid-5B*	BS00097030_51	5B	684349754	foggia	4,19-6,05	4,95-6,88	2,63-10,13	1,2,4
					metaponto	5,85-6,97	6,68-7,83	11,05-18,07	1.3
					combined	3,78-12,47	4,52-13,46	5,48-10,36	1,3,5
Kernel length	*Q.KerLen-2B.1*	BobWhite_rep_c65414_125	2B	728962900	foggia	4.04	4.79	8.93	1
					metaponto	4,66-7,49	5,45-8,37	8,69-12,69	1,2,4
					combined	3,59-7,77	4,32-8,65	11,83-13,75	1.4
	*Q.KerLen-2B.2*	Kukri_c74165_204	2B	682099186	foggia	3,65-7,87	4,39-8,76	9,65-17,36	1,2,3,4
					metaponto	4,36-7,33	5,13-8,20	7,04-14,71	1,2,3,4
					combined	4,48-9,71	5,26-10,64	4,91-16,21	1,2,3,4,5
Heading date (from 1st April)	*Q.Hd-2B*	wsnp_Ex_c7003_12065828	2B	205398993	foggia	3,75-6,35	4,49-7,19	4,33-8,62	1,2,4
					metaponto	6,41-10,79	7,25-11,74	8,33-15,39	1.2
					combined	4,55-6,36	5,33-7,20	7,72-15,27	1,2,5
	*Q.Hd-3B*	RFL_Contig3455_629	3B	30171169	foggia	4.90	5.69	3.41	5
					metaponto	4,97-5,63	5,77-6,45	3,09-6,83	1,2,5
					combined	3,03-3,67	3,73-4,40	2,47-11,93	1.2
	*Q.Hd-5B*	Ra_c73292_443	5B	511520054	foggia	3,58-8,69	4,31-9,60	5,12-20,36	1,2,4
					metaponto	3,02-5,81	3,71-6,64	4,39-9,29	1.2
					combined	3.57	4.3	6.35	1
	*Q.Hd-6B*	Tdurum_contig10194_765	6B	579819162	foggia	7.20	8.07	7.96	5
					metaponto	3.14	3.85	2.26	4
					combined	8.96	9.87	8.41	5
	*Q.Hd-7B*	Kukri_c14766_484	7B	617849760	foggia	12.20	13.18	17.1349	5
					metaponto	5,68-13,28	6,51-14,27	12,04-18,79	5.4
					combined	3.03	3.73	6.71	5
Blackstain Blackpoints	*Q.Bp-5A*	Ra_c69221_1167	5A	43344260	foggia	4.43E+00	5.20	0.00	5
					metaponto	4.01	4.76	10.16	1
					combined	-	-	-	-
Flag leaf: glaucosity of blade (lower side)	*Q.FGBG-2B*	RAC875_c34512_685	2B	5433774	foggia	4.96	5.75	12.94	5
					metaponto	4,87-7,05	5,66-7,92	8,69-21,72	1,2,5,4
					combined	4,72-6,28	4,25-7,12	11,94-18,24	1.5
Flag leaf: glaucosity of sheath	*Q.FGSG-2B.1*	RAC875_c34512_685	2B	5433774	foggia	3.24	3.95	11.86	5
					metaponto	5,78-8,07	6,60-8,96	20,17-28,92	1,2,3,5,4
					combined	4,86-8,93	5,65-9,85	10,87-22,46	1,2,4,5
	*Q.FGSG-2B.2*	BS00023068_51	2B	7492031	foggia	3.95	4.70	6.00	3
					metaponto	3.15	3.85	8.84	1
					combined	3,14-4,23	3,84-4,99	2,97-6,74	1,3,5
Kernel: coloration with phenol	*Q.KerPhe-2A*	Ku_c13700_1196	2A	706929635	foggia	5,61-14,51	6,43-15,53	15,31-37,55	1,2,3
					metaponto	8,47-10,66	9,37-11,61	4,41-44,21	1,2,3
					combined	3,21-7,02	3,92-7,89	17,19-22,60	1,4,6
	*Q.KerPhe-4B*	BS00021984_51	4B	33909315	foggia	3.61	4.34	7.32	1
					metaponto	5,03-5,94	5,82-6,77	2,13-3,15	1.3
					combined	-	-	-	-
	*Q.KerPhe-3A*	AX-95195012	3A	17799659	foggia	5.91	6.74	17.19	1
					metaponto	3,12-4,34	3,82-5,11	4,71-4,93	2,3,4
					combined	-	-	-	-
Kernel: length of brush hair in dorsal view	*Q.KerBrLen-2B*	BobWhite_c7145_355	2B	10451907	foggia	3.09	3.79	4.12	5
					metaponto	3,28-4,07	3,99-4,83	6,72-7,16	3.4
					combined	3.39	3.81	6.53	4
Spike: colour (at maturity)	*Q.SpkCol-2B*	Excalibur_c34937_710	2B	6711112	foggia	5,77-6,05	6,59-6,89	22,70-27,92	1.2
					metaponto	5,30-7,15	6,11-8,02	4,73-18,18	1,2,5,4
					combined	4,74-9,35	5,53-10,27	13,15-24,18	1,2,3
	*Q. SpkCol-3B*	BS00065107_51	3B	663563578	foggia	4.98	5.78	7.68	4
					metaponto	5.02	5.81	3.56	5
					combined	-	-	-	-
Spike: shape	*Q.Ss-3B*	BS00087278_51	3B	575368729	foggia	3.38	4.10	4.70	5
					metaponto	3.11	3.81	3.70	5
					combined	-	-	-	-
Lower glume: shape (spikelet in mid-third of ear)	*Q.Ls-7B*	BS00101364_51	7B	667477468	foggia	3,77-5,07	4,51-5,87	9,84-17,73	1,2,3,4,5
					metaponto	3.97	4.72	6.12	5
					combined	-	-	-	-
Lower glume: pubescence of external surface (spikelet in mid-third of ear)	*Q.Lp-5B*	Tdurum_contig98569_56	5B	585859189	foggia	4.17	4.93	7.98	5
					metaponto	3,03-3,85	3,72-4,59	7,53-7,59	3.5
					combined	3.79	3.98	7.67	5

For each QTN, the SNP ID, chromosome, position, environment, LOD score,-log10(P), r^2^ (%), and GWA method are reported.

The twenty-eight QTNs were distributed on eleven chromosomes: 2A, 2B, 3A, 3B, 4A, 4B, 5A, 5B, 6A, 6B, and 7B. The highest number of QTNs was found for HD (five QTNs), followed by TCC and KerPhe (three QTNs), and PH, KerLen, FGSGls and SpkCol (two QTNs each). All the remaining traits had only a QTN. Among QTNs associated with HD, three of them, on chr. 2B, 5B and 7B, were major (R^2^ ≥ 10% at least in one environment), whereas the remaining two were minor (**Table 1**). Instead, all QTNs associated with TCC (on chr. 2A and 6B), KerLen (on chr. 2B), KerWid (on chr. 5B), and Yber (on chr. 6A) were major since they explained the phenotypic variance > 10% in at least one environment. Furthermore, six QTNs (for FGBGls, FGSGls, KerPhe, SpkCol and GluShp) were major, whereas the remaining were minor. By contrast, QTNs for KerAre (on chr. 3B) and BP (on chr. 5A) were minor. To further strengthen our results, BLUPs values were calculated within both environments and tested as input for ML-GWAS. All reliable QTNs were confirmed by BLUP values except GluShp, SpkShp and BP, and for this reason, they were discarded from further analysis. Seventeen QTNs were confirmed by at least two different models when BLUPs values were used as input (**Table 1**). The *Q.Kcp-4B*, *Q.Kcp-3A* and *Q.Sc-3B* were not confirmed with BLUPs. The *Q.Hd-5B, Q.Hd-6B* and *Q.Hd-7B* were also confirmed by BLUPs, although these associations were found by a single multi-locus model.

We have also identified some QTN clusters for different traits (**Figure 1B**). For example, the *Q. FGBGls -2B*, *Q.FGSGls-2B*, *Q.SpkCol-2B* and *Q.KerBrLen-2B* co-localized on the short arm of chromosome 2B, suggesting that they were not distributed evenly in the wheat genome, but they tended to cluster chromosome regions. A genomic region (706-743 Mb) on chr. 2A was associated both with TCC and KerPhe.

### LD-haplotype blocks within LR, OC and MC

**Figure 2:**
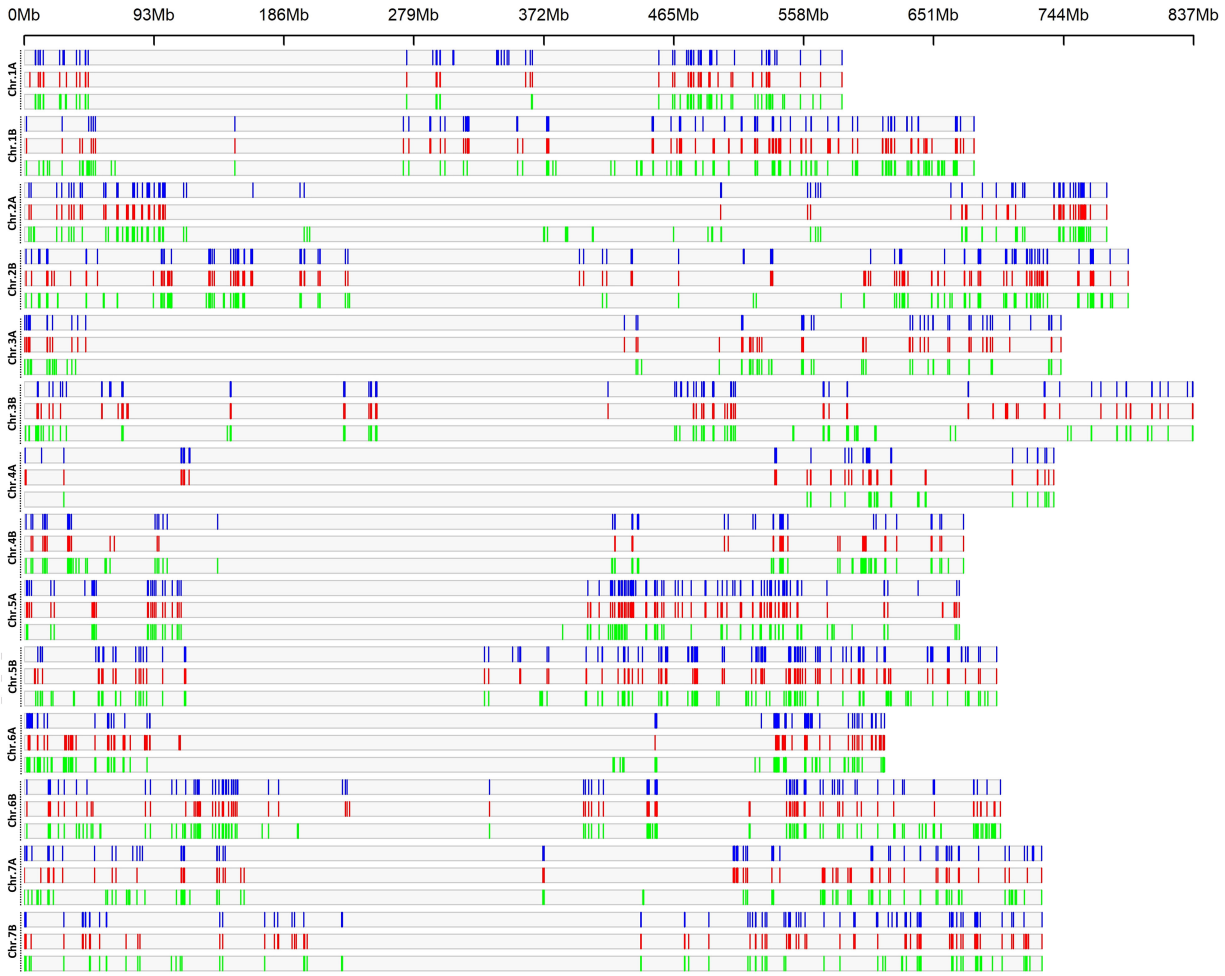
LD haplotype block density plot chromosome wise within 1 Mb window size. The horizontal axis shows the chromosome length (Mb); the different color depicts LD block density for landraces (blue), old (red) and modern (green) cultivars.

Analysis of the LD haplotype block detected 1,691, 1,778 and 1,961 SNPs in 580, 585 and 612 haplotype blocks across the genomes, for LR, OC and MC, respectively (**Figure 2**; **Tables S4**, **S5**); the blocks were distributed according to the length of each chromosome, with the highest numbers on B genome (931, 990 and 1126 for LR, Ocs and MC, respectively) than in A genome (760, 788 and 835 for LR, Ocs and MC, respectively). The highest number of haplotype blocks was detected on chromosomes 5B (66), 2B (65) and 2A (66) for LR, Ocs and MC, respectively, whereas the lowest number was on chromosome 4A for all populations. The total length of blocks was highest in MC (685.71 Mb) as well as the mean length of blocks.

A total of 54, 36 and 136 LD blocks were specific for the LR, OC and MC groups and were distributed on all chromosomes, except for chr. 3A and 7A in OC. The longest blocks were of 3.68 Mb (chr. 5B), 3.72 Mb (chr. 2B) and 3.93 Mb (chr. 1B) for LR, OC and MC, respectively (**Table S5**).

**Figure 3:**
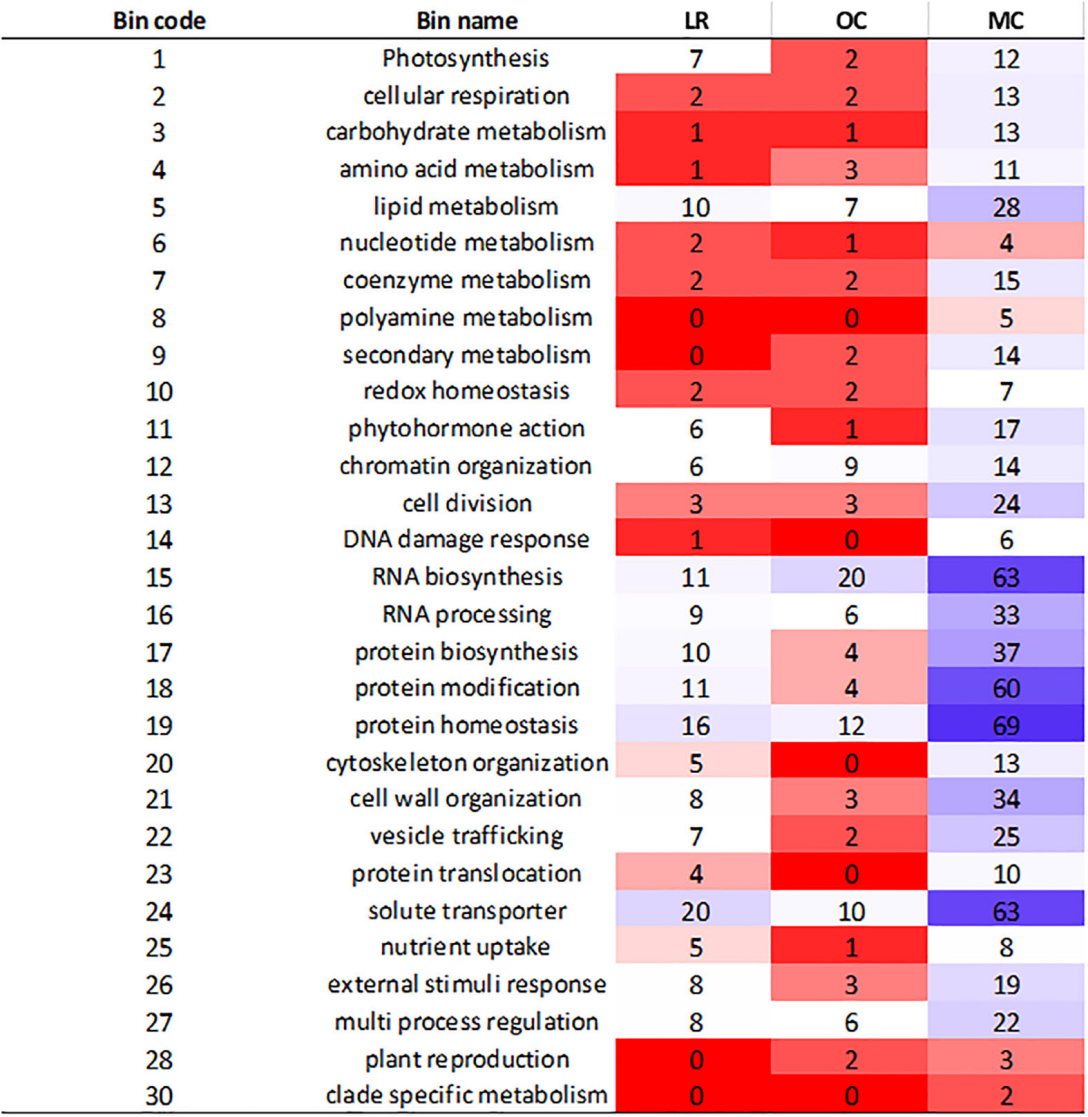
Heatmap illustrating the classification of genes in BIN code. The genes were selected in the specific LD haplotype blocks for landraces (LR), old (OC) and modern (MC) cultivars. Category number 35, “not assigned”, is not shown, and contained more than 50% of the assignments.

The analysis of genes within LD haplotype blocks showed important differences between the three groups. We retrieved 2,285 functionally characterized genes, out of which 425, 251 and 1,609 were specific to LR, OC and MC, respectively. To further understand their biological role, they were categorized in MapMan (**Figure 3**). After mapping, 165 (LR), 108 (OC) and 644 (MC) genes were assigned to different bins, related to specific functional categories (**Figure 3**). The main subclasses of metabolism-related genes were associated with RNA biosynthesis, RNA processing, protein modification, protein homeostasis and solute transporter. Most of them belonged to the modern cultivars. In contrast, polyamine metabolism, secondary metabolism, DNA damage response, cytoskeletal organization, protein translocation, and plant reproduction were poorly or absent in LR and OC.

To provide additional insight into the genes within LD blocks, we performed GO enrichment analysis separately for LR, OC and MC (**Table S6**). GO results showed that 26 and 12 genes belonging to the same biological process (response to endogenous stimulus, GO:0009719) were significantly enriched in LR and OC, respectively, although they were located on different chromosomes. In detail, the genes were mapped on chr. 1A, 5B and 7B for the LR, and on chr. 2A, 2B and 4B for the OC. All genes in LR were classified as “responses to auxin” (ancestor GO:0009733), while the genes analyzed in OC showed different ancestor GO terms (**Table S6**). Most genes were grouped in three large blocks, out of which two were on chr. 5B (block 371, 1.1 Mb) and 7B (block 562, 2.5 Mb) in LR, and one (block 169, 3.2 Mb) on chr. 2B in OC. These genes annotated as “saur-like auxin-responsive” and “btb/poz and math domain-containing protein” were organized in clusters. Other genes related to ethylene signaling and response pathway were in linkage with Cytochrome P450 and WD-repeat proteins on chr. 2A (blocks 106 and 120) in OC.

In addition, the GO term “response to stimulus” (GO:0050896) was also enriched in LR. Many “NBS-LRR disease resistance proteins” were in linkage with CLAVATA3/ESR (CLE)-related protein and Photosystem I reaction center subunit III, as well as genes related to abiotic stresses and adaption (telo2-interacting protein, Ribulose-phosphate 3-epimerase, Peroxidase, dna-3.methyladenine glycosylase) were found in blocks with genes involved in plant growth and development such as 26S proteasome non-ATPase regulatory and nac domains.

In MC, thirty-five genes were related to the biological function “enzyme regulator activity” (GO:0030234) and were localized on chr. 1B, 2A, 3A, 3B, 4B, 5B, 6A, 6B and 7A. The main ancestor GO Term were associated with enzyme inhibitor activity and defense response. The genes included clusters of disease resistance protein (TIR-NBS-LRR, RPM1) in tandem with Phloem protein 2 genes (block 35 on chr.1B), and Plant Invertase/Pectin Methylesterase Inhibitor, serpin protein and Chymotrypsin inhibitor organized in large blocks. A cluster of Chymotrypsin inhibitor genes was in linkage with the frigida and Growth-regulating factor genes on chr. 7A (block 537) known as key genes in flowering time. In addition, other genes related to the spikelets per spike phenotype (APO, chr. 6A) and photoperiod and phototropism such as radialis, Phytochrome kinase (PKS), nonphototropic hypocotyl 3 (NPH3, TRITD6Av1G011830) were found.

### QTNs in LD haplotype blocks

We searched candidate genes associated with significant QTNs using LD blocks and LD decay. A total of eight QTNs for LR and MC and five for OC were included in 21 haplotype blocks, of which three were shared among the three populations (*Q.Ka-3B*, *Q.Lp-5B and Q.Ls-7B*) (**Table S7**). LR and MC had two haplotype blocks in common (*Q.Klh-2B*, *Q.Hd-3B*), while one was found between LR and OC (*Q.Kcp-4B*). Among these, the largest haplotype block was detected in LR (2870.68 kb) on chr. 7B, whereas the lowest (0.03 kb) in LR on chr. 5A. A variation in size, as well as in frequency of the haplotypes was observed for the three blocks including the QTNs *Q.Ka-3B*, *Q.Lp-5B* and *Q.Ls-7B*. The first had a lower size in LR (647.549 kb) than in OC and MC (1904.04 kb), and also the alleles and their frequencies changed over the three populations (**Table S7**). For the second, the size, alleles and frequencies slightly changed moving from LR to OC. For the third, a decrease in size was observed from LR (2870.68) to OC and MC (67.596), with a change in allele and frequencies. The most significant candidate genes associated with QTNs identified In this study were reported in **Table 2**.

**Table 2:** List of the most important candidate genes identified in QTN flanking regions.

Trait	QTN	SNP ID	Chr	Gene position	Transcript ID	Gene
	*Q.Ph-2B*	Kukri_c36879_83	2B	97248107-97248499	TRITD2Bv1G039250	cell elongation protein/DWARF1/DIMINUTO
Plant height				100468124-100468429	TRITD2Bv1G040470	Flowering-promoting factor 1-like protein
				201182907-201184571	TRITD2Bv1G076890	GRAS transcription factor
	*Q.Ph-4A*	Tdurum_contig31218_344	4A	733395300-733401316	TRITD4Av1G262670	RING/FYVE/PHD zinc finger
				733426625-733430279	TRITD4Av1G262700	WD repeat-containing protein
Kernel area	*Q.KerAre-3B*	RAC875_rep_c83245_239	3B	486046362-486049800	TRITD3Bv1G158120	Glycosylphosphatidylinositol anchor attachment
				484788708-484798884	TRITD3Bv1G157650	Glutamate synthase (GOGAT)
Yellow berry	*Q.Yber-6A*	Excalibur_c9713_247	6A	46078171-46078562	TRITD6Av1G019550	NADH-ubiquinone oxidoreductase subunit
Total Carotenoid Content	*Q.Tcc-2A*	BobWhite_c31163_694	2A	742575242-742579242	TRITD2Av1G279100	Lipoxygenase
				743684815-743703529	TRITD2Av1G279520	Epoxide hydrolase 2
				745833575-745834999	TRITD2Av1G280680	scarecrow-like transcription factor SlSCL3
	*Q.Tcc-6B.2*	Tdurum_contig11700_1247	6B	132017865-132020956	TRITD6Bv1G046390	Squamosa promoter-binding
				132021841-132051890	TRITD6Bv1G046410	Terpene cyclase/mutase
				132618310-132622371	TRITD6Bv1G046670	GDSL esterase/lipase
	*Q.Tcc-6B.1*	BobWhite_c1633_643	6B	645615422-645616174	TRITD6Bv1G208120	Aspartate aminotransferase
				644455549-644460412	TRITD6Bv1G207740	Zinc finger protein VAR3
Kernel width	*Q.KerWid-5B*	BS00097030_51	5B	683865860-683866078	TRITD5Bv1G246130	cell elongation protein/DWARF1/DIMINUTO
				683864683-683867193	TRITD5Bv1G246120	acyl-CoA-binding
Kernel lenght	*Q.KerLen-2B.1*	BobWhite_rep_c65414_125	2B	729800488-729800799	TRITD2Bv1G241110	geranylgeranyl pyrophosphate synthase 3
Heading date (from 1st April)	*Q.Hd-2B*	wsnp_Ex_c7003_12065828	2B	201182907-201184571	TRITD2Bv1G076890	GRAS transcription factor
				205218481-205219553	TRITD2Bv1G078080	BES1/BZR1-like protein 1
				206152169-206152510	TRITD2Bv1G078550	SAUR-like auxin-responsive protein family, putative
	*Q.Hd-3B*	RFL_Contig3455_629	3B	30170900-30173747	TRITD3Bv1G013460	E3 ubiquitin-protein ligase
Flag leaf: glaucosity of blade (lower side)	*Q.FGSG-2B.1*	RAC875_c34512_685	2B	8627534-8630564	TRITD2Bv1G004160	Fructokinase-2
Flag leaf: glaucosity of sheath	*Q.FGSG-2B.2*			3016760-3023102	TRITD2Bv1G001660	O-acyltransferase WSD1
Kernel: coloration with phenol	*Q.KerPhe-2A*	Ku_c13700_1196	2A	706301918-706306994	TRITD2Av1G261300	Ppo-A1
				706523800-706525741	TRITD2Av1G261390	Ppo-A2
	*Q.KerPhe-4B*	BS00021984_51	4B	29292990-29294855	TRITD4Bv1G012280	Della protein GAI
			4B	31293156-31294696	TRITD4Bv1G013070	CONSTANS-like zinc finger protein

## Discussion

Improving grain production while maintaining environmental sustainability under climate change remains the main breeding goal in the coming years, especially in vulnerable environments, such as the Mediterranean Basin (Ceglar et al., 2021). Understanding the effects produced by durum wheat breeding activities on the main traits of agronomic interest could help the selection of new varieties to face climatic conditions and better exploit the genetic variability of this species (De Vita and Taranto, 2019). Dissecting the genetic architecture of complex quantitative traits using high-density SNP markers in wheat could have practical implications in durum wheat molecular breeding for improving yield potential and grain quality but also for providing tools to accelerate plant variety protection and registration (Arriagada et al., 2020; Mulugeta et al., 2023).

In the current study, we exploited the ML-GWAS approach to study the genetic basis of 34 morpho-phenological and agronomic traits and identify QTNs to be exploited for marker-assisted selection and/or in UPOV protocol for durum wheat varietal protection. In addition, we defined haplotype blocks, using the LD-based approach (Qian et al., 2017), to identify genomic regions and/or candidate genes associated with agronomic traits under study and the impact of breeding programs on their architecture.

Most of the traits investigated in our panel showed normal distributions, typical of traits controlled by multiple QTLs and highly vulnerable to environmental factors. The six ML-GWAS methods confirmed the genetic complexity, revealing different traits-associated QTNs across all wheat chromosomes. The BLUP prediction method, a parameter to minimize the environmental bias allowing the estimation of the true individual genetic value (Robinson, 1991), increased the consistency of the six ML-GWAS models reducing the number of associated QTNs. Among all models used, the pLARmEB detected the highest number of associations, confirming previous studies on soybean (Zhang et al., 2018; Fang et al., 2020), maize (An et al., 2020), rape (Khan et al., 2019) and common bean (Delfini et al., 2021).

Comparing our GWAS findings with those of previous studies, we found that some important genes controlling flowering time and plant height in durum wheat were in LD decay with QTNs identified in this study. The *Q.Ph-2B* (*Kukri_c36879_83*) was in LD decay with candidate genes affecting PH such as diminuito/dwarf1 gene, Flowering-promoting and gras transcription factors involved in steroid synthesis (Klahre et al., 1998) and in gibberellin signal transduction pathways, respectively (Kovi et al., 2011; Cheng et al., 2022). In the same region, Maccaferri et al. (2011) found a QTL for HD. The *Q.Hd-7B* was identified in the proximity of QTLs for HD and PH previously identified by Maccaferri et al. (2014). The MTA *Ra_c73292_443*, associated with the heading date, was also found as a marker harboring the 5B.3 hotspot able to differentiate wheat Mediterranean landraces based on adaptative traits (i.e., flowering time) (Yannam et al., 2023).

Several QTNs found in the present study were related to kernels, traits usually poorly investigated in the literature. Among these, the vitreousness of the kernels is one of the grain qualitative parameters most appreciated by the pasta industry and the market. Vitreous kernels are believed to have higher protein content and to have higher grain quality, compared to non-vitreous kernels, also called ‘yellow berry’ (Dexter et al., 1988; Dexter et al., 1989; Samson et al., 2005; Sieber et al., 2015; Fu et al., 2018). Yellow berry is expressed by the presence of starchy spot areas in a usually vitreous grain conferring a less compact structure with numerous open spaces and physically discontinuous protein matrix in kernels (Dexter et al., 1989, Turnbull and Rahman, 2002; Samson et al., 2005). As a result, the yellow berry is a primary factor in the marketing of durum, influencing the milling and end-product quality of durum wheat. Poor nitrogen availability is considered the most critical factor in the determination of yellow berries (Morris and Beecher, 2012), however, few genetic studies have been conducted to elucidate the genetic basis of this trait in wheat. We found a strong association for yellow berry trait on chromosome 6A (*Q.Yb-6A*) in a region containing a nadh-ubiquinone oxidoreductase gene, involved in starch metabolism in rice (Hu et al., 2018). This gene could be considered a good candidate to be explored to understand its role in wheat. Previously, Pshenichnikova et al. (2008) detected QTLs for grain vitreousness on chromosomes 3A, 5D, and 6A using the bread wheat ITMI population. Dhaliwal et al. (1986) studied the inheritance of yellow berry among the progeny of six bi-parental crosses, and the Chinese Spring monosomics CIMMYT breeding line had the major dominant genes on chromosomes 1A and 7A. Prashant et al. (2011) mapped the yellow berry on chromosomes 5D and 2D in a genetic linkage map of hexaploidy wheat using SSR markers. Two QTLs for yellow berry tolerance were also reported by Ammiraju et al. (2002) in a bread wheat RIL population on chromosomes 5D and 6B, respectively. Thus, our result may support the fine mapping to localize the candidate gene responsible for yellow berry in durum wheat.

The *MTA Ra_c69221_1167* (*Q.Bp-5A*) at 43.43 Mb on chr. 5A, associated with the black point disease resistance, was previously reported as an important player conferring resistance to Septoria and Powdery Mildew in winter wheat (Alemu et al., 2021). Our *Q.Bp-5A* appears to be different from those previously mapped to the same chromosome by other authors in bread wheat. Li et al. (2022) identified two QTLs for black point resistance on chr. 4A and 5A (2.1-274.2 Mb), explaining 3.3%-15.1% of the phenotypic variances, respectively. The *QBp.caas-5AS* for black point resistance detected by Liu et al. (2016) was at 110.7 Mb. Another six loci for black point resistance were detected on chromosome 5A but none overlapped with our *Q.Bp-5A* (Li et al., 2020; Lv et al., 2020).

Several phenotypic traits considered in this study are part of the UPOV technical protocols and used for DUS examination of candidate varieties for registration in the National/European catalogue. Currently, the UPOV test guidelines for numerous crops, describe the relevant traits to be evaluated, along with the recommended procedures for conducting the trials and the statistical analyses to use. In addition, UPOV guidelines establish which characteristics should be visually scored and which ones should be precisely measured. DUS testing is entirely independent from any evaluation of end-use value. UPOV characteristics for DUS testing primarily rely on morphological traits, chosen to reflect general genetic differentiation among varieties, strongly influenced by the environment and by visual assessment (Yu and Chung, 2021). The evaluation system also involves the use of a limited number of reference varieties to compare the new candidate variety. Therefore, adding the environmental influence, the operator’s subjectivity in visual scoring and the limited number of reference varieties, it seems necessary to find some alternative procedures to use in DUS testing. Since the DUS phenotypic evaluation has some limitations about the influence that environmental conditions have on the expression of these traits (Yu and Chung, 2021). The aim of the ML-GWAS analysis performed in this work was to identify QTN closely associated with DUS traits, and to integrate the use of DNA markers into UPOV test guidelines. Indeed, the analysis showed interesting results for kernel-related traits (i.e., area, length, width, coloration with phenol), flag leaf glaucosity of blade and sheath, spike color at maturity, lower glume shape, and pubescence of external surface.

Russo et al. (2014) using 136 F5 recombinant inbred lines, derived from a cross between modern durum wheat and *T*. *dicoccum*, identified six QTLs on chr. 1B, 2B, 3A, 3B, 4B and 7A, and 2 QTLs on chromosomes 3B and 4B for traits related to kernel morphology. Two QTLs reported by Russo et al. (2014) as associated with kernel length on chromosome 2B co-localized with those similar QTLs found in our study. In particular, the marker *Kukri_c21135_1071* was ~ 5 Mb from *Kukri_c74165_204* (*Q.Kl-2B.2*) and coincided also with QTL for thousand kernel weight (TKW) identified by Su et al. (2018).

The kernel coloration with phenol in durum wheat is the result of the conversion of phenol and other phenolic derivates into melanin pigments in the presence of tyrosinase (Taranto et al., 2017). Since the need to select advanced wheat breeding lines with a reduced aptitude for flour/semolina browning, a characteristic not appreciated by consumers, the kernel coloration with phenol was among the whole-seed assays also used in breeding programs to estimate the polyphenol oxidase activity (PPO) selecting those with low PPO activity in wheat (Sun et al., 2005; He et al., 2007; Taranto et al., 2015). Our results confirmed what was previously reported by Zhai et al. (2020) and Taranto et al. (2021; Taranto et al., 2022). The *Q.Kcp-2A* harbors the PPO locus, with the marker *Ku_c13700_1196* in LD decay with *Ppo-A1* and *Ppo-A2* genes, confirming the markers associated with this trait as one of the most suitable genetic models to support DUS phenotypic protocols.

Unlike the previous one, which was already known in the literature, the associations found for flag leaf gloucosity (both lamina and sheath) represented a new result in durum wheat. In fact, the *Q.FGSGls-2B.1* identified by the *RAC875_c34512_685* marker mapped less than 1 Mb from the *Epistatic inhibitors of glaucousness* (*Iw1*) locus previously described by Yoshiya et al. (2011) in durum wheat using SSR markers and cloned by Huang et al. (2017) in bread wheat. However, it remains that, the chromosome region identified remains a valid result to be validated with further investigations.

In our previous study (Taranto et al., 2020) we compared the patterns of genetic variation observed in the Italian durum wheat germplasm, including LR, OC and MC, in order to better understand the effect of artificial selection and provide a list of genes/loci under selection associated with useful agronomic traits. In the present work, we corroborated the observed signature of selection using the LD haplotype blocks (HBs) partitioning, through the evaluation of haplotype diversity (i.e. allele type, frequency, length) within haploblocks across LR, OC and MC. The results revealed large and significant differences in the extent and pattern of LD among the three groups. As expected, moving from LR to MC, the number and size of LD HBs increased, confirming the increasing LD decay previously reported by Taranto et al. (2020). The observed pattern of LD decay was in line with the estimation over three main breeding periods (1915-1979, 1980-1999 and 2000-2020) made by Roncallo et al. (2021) in a worldwide durum wheat collection. The higher haplotype diversity as well as the higher number of haplotypes in MC than OC and LR indicated that modern breeding practices directly affected the composition and variation of gene pools over generations, diminishing effective population sizes, increasing inbreeding, and consequently increasing LD and HBs. The longest blocks were on chromosomes 1B, 2B and 5B for LR, OC and MC, respectively. Long-range LD blocks on 1B were also reported by Joukhadar et al. (2019) and Roncallo et al. (2021) and signatures of selection based on LD on 1B were observed by Maccaferri et al. (2014) confirming the selective pressure on genomic loci related to grain quality traits. The identification of different HBs among landraces and modern cultivars affecting agronomic performance within QTL hotspots were also reported by Royo et al. (2021) and Soriano et al. (2021).

A likely cause of the observed differences in length and number of HBs among the three genetic groups could be related to the breeding effect exerted on genes involved in response to abiotic and biotic stresses and gravitropism and phototropism; these latter are considered an adaptive response of crucial importance in plants (Lariguet et al., 2006; Kippes et al., 2020).

An enrichment of *saur-like auxin-responsive* genes was found in the HBs of LR and OC, although on different chromosomes. These are a family of auxin-responsive genes involved in plant growth and in response to internal and external signals (Stortenbeker and Bemer, 2019). It has been demonstrated that the overexpression of SAUR66-5B can increase the biomass and grain yields of transgenic wheat, as well as the nitrogen concentration and accumulation (Lv et al., 2022). In addition, the role in abiotic stress signaling of SAUR genes in wheat was also elucidated by Abhinandan et al. (2018). Our findings showed an enrichment of key genes as signal transducers in phototropism signalling in the HBs specific for MC. In particular, we found the phototropic-responsive genes PKS and NPH3, for which the role in *Arabidopsis* and cotton phototropism has been demonstrated (Lariguet et al., 2006; Christie et al., 2018; Grover et al., 2020; Kippes et al., 2020; Kimura et al. 2022). These genes could be good candidates to better understand the adaptative mechanisms moving from landraces to modern cultivars, together with the most renowned genes that we found in several HBs and QTNs such as *TaGW2* genes (6A and 6B, Qin et al., 2014), *Ppo* (Taranto et al., 2021), *Ppd* (Arjona et al., 2018), dwarfism genes *Rht-1* (Pearce et al., 2011), and *Vrn* (Royo et al., 2020).

The fact that most of the genes included in HBs are involved in adaptative mechanisms, highlighted the necessity to explore their allelic composition in the wild, domesticated, and modern germplasm with the aim to broaden the genetic variability and constitute new ideotypes to be used in durum wheat breeding programs.

## Conclusions

Several phenotypic traits considered in this study are part of the international legal framework established by the UPOV guidelines for awarding Plant Breeders’ Rights PBR, similar to patent or intellectual property rights to new crop varieties. For some of them (i.e., kernel coloration with phenol and flag leaf glaucosity), strong associations were found through GWAS and LD haplotype block analysis, proving their power in detecting genetic variants associated with the traits of interest and for evaluating the impact of breeding programs. In particular, the increase in the number and length of haplotype blocks in MC suggested the high selective pressure exerted by breeders for the traits of agronomic interest.

We believe that the results reported here will not only expand the knowledge regarding the genetic architecture of many of these traits but also allow the employment of diagnostic/perfect genetic markers in current UPOV descriptive protocols, strongly influenced by environment and visual assessment, thus improving the plant variety protection and registration system.

## Data availability statement

Publicly available datasets were analyzed in this study. This data can be found here: Figshare repository (doi: 10.6084/m9.figshare.11835663).

## Author contributions

FT and PV conceived the work, coordinated and supervised the activities. FF, SR, MS, LG and GT phenotyped the genetic materials. FT and SE performed statistical, QTNs and LD block analyses and data visualization. PV, FT, and SE contributed to interpretation and wrote the manuscript. All authors critically revised the manuscript. All authors read and approved the final manuscript.

## Supplementary material

Supplementary Figure 1 Frequency distribution of BLUPs for the 34 traits among cultivars. X-axis and Y-axis show BLUP values and the frequency, respectively.
