# Supplementary figures and images for: Breeding effects on durum wheat traits detected using GWAS and haplotype block analysis
Source: Front Plant Sci. 2023 Sep 19;14:1206517. doi: 10.3389/fpls.2023.1206517 (PMC10546023; doi:10.3389/fpls.2023.1206517)

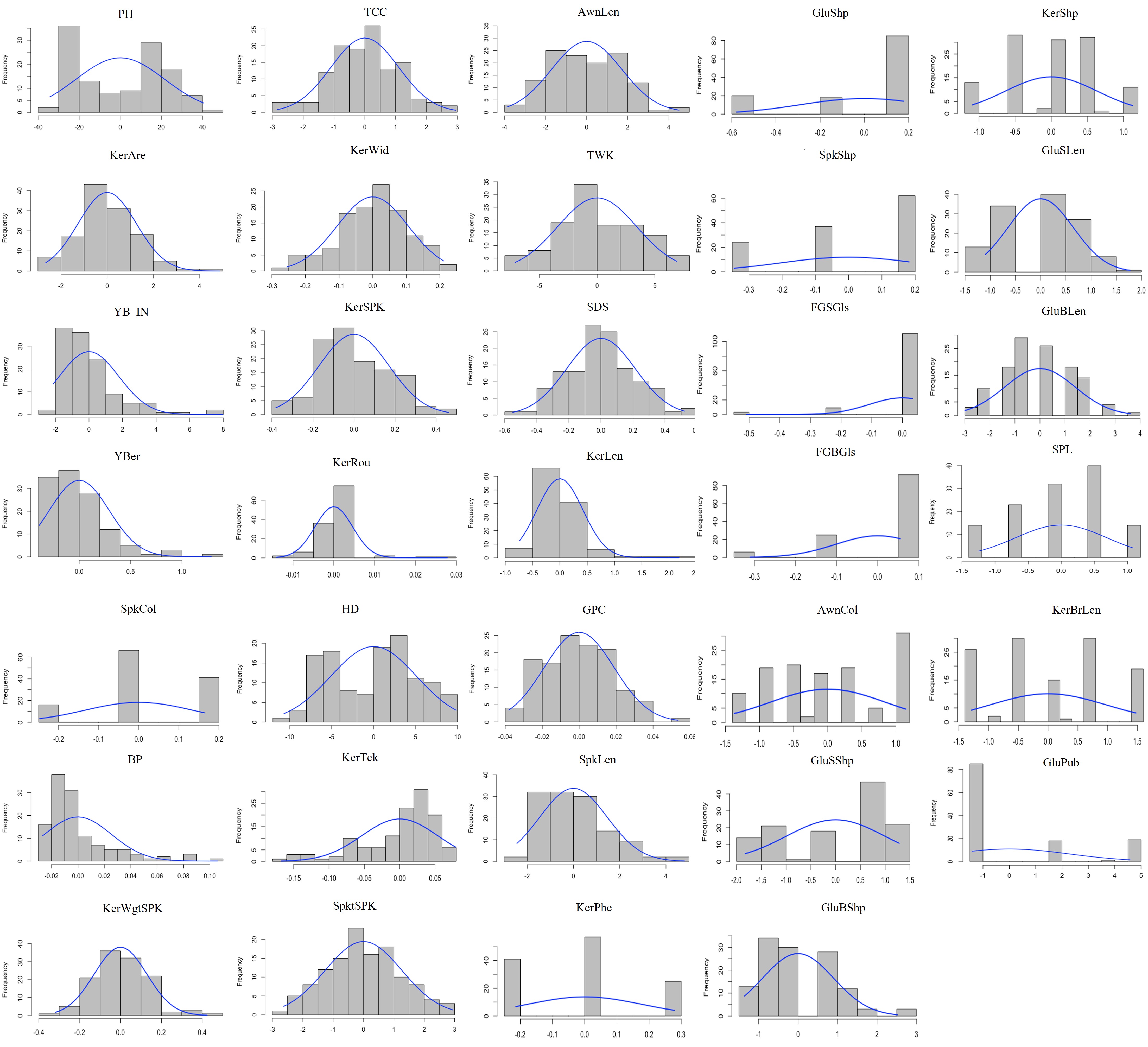

Supplement: Supplementary Figure 1 — Frequency distribution of BLUPs for the 34 traits among cultivars. X-axis and Y-axis show BLUP values and the frequency, respectively. [file Image_1.jpeg]
